# Vaccination Coverage for Selected Vaccines, Exemption Rates, and Provisional Enrollment Among Children in Kindergarten — United States, 2016–17 School Year

**DOI:** 10.15585/mmwr.mm6640a3

**Published:** 2017-10-13

**Authors:** Ranee Seither, Kayla Calhoun, Erica J. Street, Jenelle Mellerson, Cynthia L. Knighton, Ashley Tippins, J. Michael Underwood

**Affiliations:** ^1^Immunization Services Division, National Center for Immunization and Respiratory Disease, CDC; ^2^Association of Schools and Programs of Public Health Fellowship, Immunization Services Division, National Center for Immunization and Respiratory Disease, CDC.

State and local school vaccination requirements help protect students and communities against vaccine-preventable diseases (*1*). CDC reports vaccination coverage and exemption data for children attending kindergarten (kindergartners) collected by federally funded immunization programs in the United States.* The typical age range for kindergartners is 4–6 years. Although vaccination requirements vary by state (the District of Columbia [DC] is counted as a state in this report.), the Advisory Committee on Immunization Practices recommends that children in this age range have received, among other vaccinations, 5 doses of diphtheria, tetanus, and acellular pertussis vaccine (DTaP), 2 doses of measles, mumps, and rubella vaccine (MMR), and 2 doses of varicella vaccine (*2*). This report summarizes 2016–17 school year MMR, DTaP, and varicella vaccination coverage reported by immunization programs in 49 states, exemptions in 50 states, and kindergartners provisionally enrolled or within a grace period in 27 states. Median vaccination coverage^†^ was 94.5% for the state-required number of doses of DTaP; 94.0% for 2 doses of MMR; and 93.8% for 2 doses of varicella vaccine. The median percentage of kindergartners with an exemption from at least one vaccine^§^ was 2.0%, similar to 2015–16 (1.9%). Median grace period and provisional enrollment was 2.0%. Vaccination coverage remains consistently high and exemptions low at state and national levels. Local-level vaccination coverage data provide opportunities for immunization programs to identify schools, districts, counties, or regions susceptible to vaccine-preventable diseases and for schools to address undervaccination through implementation of existing state and local vaccination policies (*1*) to protect communities through increased coverage.

Federally funded immunization programs partner with departments of education and school nurses and other school personnel to assess vaccination coverage and exemption status of children enrolled in public and private kindergartens.^¶^ In accordance with state and local school entry requirements, parents and guardians submit their children’s vaccination records or exemption forms to schools, or schools obtain kindergartners’ records from their states’ immunization information systems. During the 2016–17 school year, 49 states reported data on coverage for all state-required vaccines among public and private school kindergartners, 50 states reported exemption data on public school kindergartners, and 49 states reported exemption data on private school kindergartners. Seven states reported coverage and exemption data for at least some homeschooled kindergartners.** Twenty-seven states reported data on kindergartners who, at the time of the assessment, were attending school under a grace period (a set number of days during which a student can be enrolled and attend school without proof of complete vaccination or exemption) or provisional enrollment (a provision that allows a student without complete vaccination or exemption to attend school while completing a catch-up vaccination schedule).

During the 2016–17 school year, vaccination assessments varied by immunization program because of differences in state requirements regarding required vaccinations and number of doses required, vaccines assessed, school assessment, data reported, and available resources. Among the 50 states reporting data, 35 used a census to collect kindergarten vaccination data; nine used a sample; four used a voluntary school response; and two used a mix of sampling methods.^††^ States used the same methods to collect both vaccination coverage and exemption data, except in Alaska, Kansas, Virginia, and Wisconsin, where a sample was used to collect vaccination coverage data and a census to collect exemption data. Five states (Delaware, Hawaii, Nevada, New Mexico, and South Carolina) used a sample for both vaccination coverage and exemption data. Kindergartners were considered up-to-date and included in the coverage estimate for a given vaccine if they received all doses required for school entry,^§§^ except in seven states^¶¶^ that considered kindergartners up-to-date only if they had received all doses of all vaccines required for school entry in those states. Kindergartners with a history of varicella disease were reported as either vaccinated against varicella or medically exempt, varying by immunization program. Medical exemptions were issued by a health care provider; all other exemptions (i.e., religious and philosophical) were nonmedical.

This report presents vaccination coverage for MMR, DTaP, and varicella vaccines. Coverage for these vaccines and hepatitis B and poliovirus vaccines that are required in most states is presented on SchoolVaxView (*3*). Vaccination coverage and exemption estimates were adjusted based on survey type and response rates.*** Medians and ranges of state MMR vaccination coverage and of exemption rates collected from the 2011–12 school year through the 2016–17 school year were examined over time. During the 2016–17 school year, vaccination coverage data were reported for approximately 3,973,172 kindergartners, exemption data for approximately 3,666,870, and grace period and provisional enrollment data for approximately 2,463,131.^†††^

Since the 2011–12 school year, median kindergarten MMR vaccination coverage has remained near 95% and median exemption rates have remained ≤2% (Figure). Among the 49 states included in this analysis, median MMR coverage was 94.0% (range = 85.6% [DC] to 99.4% [Mississippi]); 20 states reported coverage ≥95%; and six states (Alaska, Colorado, Idaho, Indiana, Kansas, and DC) reported coverage <90% (Table 1). Among the 48 states that required and reported DTaP vaccination, median coverage was 94.5% (range = 82.2% [DC] to 99.6% [Maryland]); 23 states reported coverage ≥95% and six states (Alaska, Arkansas, Colorado, Idaho, Kansas, and DC) reported coverage <90%. Among the 42 states that required and reported 2 doses of varicella vaccine, median coverage was 93.8% (range = 84.6% [DC] to 99.4% [Mississippi]); 15 states reported coverage ≥95%, and seven states (Alaska, Colorado, Idaho, Indiana, Kansas, Washington, and DC) reported coverage <90%. Thirty states^§§§^ published 2015–16 or 2016–17 local-level data (county, parish, school district, school, or other level) online for vaccination coverage, exemptions, or both (Table 1).

**FIGURE:**
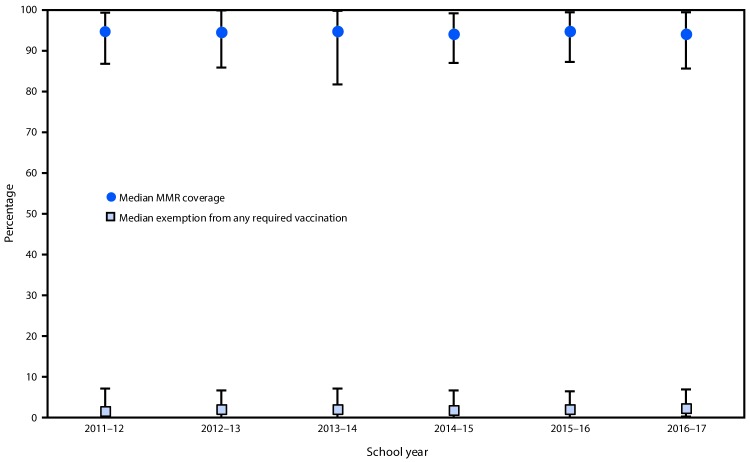
Median and range* of measles, mumps, and rubella vaccine (MMR) coverage and exemptions from any required vaccination^†^ among kindergartners — United States, 2011–12 to 2016–17 school years **Source:** School Vaccination Assessment Program, 2011–12, 2012–13, 2013–14, 2014–15, 2015–16, and 2016–17 school years. https://www.cdc.gov/vaccines/vaxview/index.html. * Data from local areas and territories are not included. Number of states whose data are included in the MMR coverage medians and ranges varied by year: 2011–12 (44 states); 2012–13 (46); 2013–14 (47); 2014–15 (50); 2015–16 (51); and 2016–17 (49). Number of states whose data are included in the exemption medians and ranges varied by year: 2011–12 through 2014–15 (46 states); 2015–16 (48); and 2016–17 (46). ^†^ Represents the number of children who are exempt from any vaccination, not just MMR.

**TABLE 1:** Estimated vaccination coverage* for MMR, DTaP, and varicella vaccines among children enrolled in kindergarten, by vaccine and immunization program — United States and territories, 2016–2017 school year

Immunization program	Kindergarten population^†^	No. (%) surveyed	Type of survey conducted^§^	Local data available online^¶^	%			
					MMR**	DTaP^††^	Varicella	
					2 doses	5 doses	1 dose	2 doses
Median^§§^					94.0	94.5	96.5	93.8
Alabama^¶¶^	58,394	58,394 (100.0)	Census	Yes	≥93.8	≥93.8	≥93.8	NReq
Alaska***^,†††^	9,815	747 (7.6)	Stratified 2-stage cluster sample	No	89.0	89.1	NA	88.9
Arizona^¶¶^	83,627	83,627 (100.0)	Census	Yes	94.0	93.9	96.7	NReq
Arkansas^§§§^	39,666	30,091 (75.9)	Voluntary response	No	91.9	89.2	NA	91.7
California^§§§^	575,305	562,924 (97.8)	Census	Yes	97.3	96.9	98.5	NReq
Colorado^¶¶^	64,440	64,440 (100.0)	Census	Yes	87.3	86.8	NA	86.1
Connecticut^¶¶^	39,002	39,002 (100.0)	Census	Yes	96.7	96.7	NA	96.5
Delaware	11,490	1,066 (9.3)	Stratified 2-stage cluster sample	No	98.5	98.7	NA	98.2
District of Columbia^¶¶^	8,522	8,522 (100.0)	Census	No	85.6	82.2	NA	84.6
Florida^¶¶,^***	224,463	224,463 (100.0)	Census	Yes	≥94.1	≥94.1	NA	≥94.1
Georgia^¶¶^	136,165	136,165 (100.0)	Census	No	≥93.3	≥93.3	NA	≥93.3
Hawaii	16,325	1,093 (6.7)	Stratified 2-stage cluster sample	No	93.5	93.3	95.3	NReq
Idaho^¶¶^	22,589	22,589 (100.0)	Census	Yes	89.9	89.8	NA	89.1
Illinois	151,309	147,857 (97.7)	Census	No	94.9	95.0	NA	94.5
Indiana	83,263	66,885 (80.3)	Voluntary response	Yes	88.9	92.1	NA	87.9
Iowa^¶¶^	39,587	39,587 (100.0)	Census	Yes	≥92.6	≥92.6	NA	≥92.6
Kansas***^,†††,§§§^	38,298	8,789 (22.9)	Stratified 2-stage cluster sample	Yes	89.5	88.7	NA	88.8
Kentucky***^,§§§^	51,487	47,814 (92.9)	Census	Yes	90.8	92.5	NA	90.4
Louisiana^¶¶^	55,257	55,257 (100.0)	Census	Yes	97.1	98.0	NA	96.5
Maine	13,834	12,462 (90.1)	Voluntary response (public), census (private)	Yes	94.9	96.3	96.7	NReq
Maryland^§§§^	71,467	70,106 (98.1)	Census	No	99.3	99.6	NA	99.0
Massachusetts^¶¶,§§§^	70,109	70,109 (100.0)	Census	Yes	96.1	96.1	NA	95.7
Michigan^¶¶^	118,777	118,777 (100.0)	Census	Yes	95.6	95.8	NA	95.3
Minnesota***	69,140	66,861 (96.7)	Census	Yes	92.8	93.2	NA	92.3
Mississippi^¶¶^	40,509	40,509 (100.0)	Census	Yes	≥99.4	≥99.4	NA	≥99.4
Missouri^¶¶^	73,355	73,355 (100.0)	Census	No	95.4	95.5	NA	95.1
Montana^¶¶^	11,956	11,956 (100.0)	Census	No	93.8	93.9	NA	92.9
Nebraska^¶¶,§§§,¶¶¶^	27,117	27,117 (100.0)	Census	No	96.7	97.2	NA	95.8
Nevada	36,885	1,348 (3.7)	Stratified 2-stage cluster sample	No	90.9	90.0	NA	90.5
New Hampshire^¶¶^	12,145	12,145 (100.0)	Census	No	≥91.5	≥91.5	NA	≥91.5
New Jersey^¶¶^	109,577	109,577 (100.0)	Census	Yes	≥96.5	≥96.5	≥96.5	NReq
New Mexico	27,119	1,214 (4.5)	Stratified 2-stage cluster sample	No	95.5	94.8	NA	94.6
New York (including New York City)^¶¶^	227,050	227,035 (100.0)	Census	Yes	97.3	96.9	NA	96.9
New York City^¶¶^	102,374	102,374 (100.0)	Census	No	97.7	97.0	NA	97.2
North Carolina***^,§§§^	126,454	111,544 (88.2)	Voluntary response	No	96.2	96.1	NA	95.9
North Dakota	9,799	9,675 (98.7)	Census	Yes	93.8	93.8	NA	93.5
Ohio	137,542	131,385 (95.5)	Census	No	92.6	92.4	NA	91.9
Oklahoma^§§§,^****	52,184	48,453 (92.9)	Census	No	NA	NA	NA	NReq
Oregon^¶¶,§§§^	45,705	45,705 (100.0)	Census	Yes	93.8	93.2	95.0	NReq
Pennsylvania	143,888	121,405 (84.4)	Voluntary response	Yes	93.6	NReq^††††^	NA	94.6
Rhode Island***^,§§§^	11,100	10,920 (98.4)	Census	Yes	95.1	95.6	NA	94.8
South Carolina	59,177	5,277 (8.9)	Stratified 1-stage cluster sample	No	96.0	96.2	NA	95.7
South Dakota	12,106	12,081 (99.8)	Census	Yes	96.7	96.4	NA	95.4
Tennessee^¶¶,^***	78,169	78,169 (100.0)	Census	No	96.9	96.8	NA	96.7
Texas (including Houston)***^,§§§^	389,999	386,149 (99.0)	Census	Yes	97.3	97.2	NA	96.6
Houston***^,§§§^	42,086	40,802 (96.9)	Census (public), voluntary response (private)	No	96.1	96.1	NA	95.7
Utah^¶¶^	49,073	49,073 (100.0)	Census	Yes	93.8	93.7	NA	94.6
Vermont^¶¶^	6,344	6,344 (100.0)	Census	Yes	93.6	93.5	NA	92.5
Virginia^†††^	102,357	4,051 (4.0)	Stratified 2-stage cluster sample	Yes	94.1	98.2	NA	92.7
Washington***	87,142	85,601 (98.2)	Census	Yes	90.5	90.8	NA	89.3
West Virginia***	28,666	19,074 (66.5)	Census (public), voluntary response (private)	No	95.9	95.7	NA	92.6
Wisconsin***^,†††,§§§^	67,607	1,472 (2.2)	Stratified 2-stage cluster sample	Yes	94.0	96.6	NA	92.8
Wyoming	NA	NA	Not conducted	No	NA	NA	NA	NA
Guam	2,703	703 (26.0)	Stratified 2-stage cluster sample	No	90.3	93.5	NReq	NReq
Marshall Islands	1,248	1,248 (100.0)	Census	No	87.3	72.8	NReq	NReq
Federated States of Micronesia (Kosrae)	194	194 (100.0)	Census	No	88.7	91.8	NReq	NReq
Federated States of Micronesia (Yap)	400	400 (100.0)	Census	No	91.3	92.0	NReq	NReq
N. Mariana Islands^¶¶^	865	865 (100.0)	Census	No	89.8	75.3	NA	88.0
Palau^¶¶,¶¶¶^	333	333 (100.0)	Census	No	59.8	64.9	NReq	NReq
Puerto Rico	23,142	1,384 (6.0)	Stratified 2-stage cluster sample	No	96.2	96.1	NA	95.9
U.S. Virgin Islands	1,244	505 (40.6)	Stratified 2-stage cluster sample	No	88.9	88.5	NA	88.1

**Abbreviations:** DTaP = diphtheria, tetanus, and acellular pertussis vaccine; MMR = measles, mumps, and rubella vaccine; NA = not available (i.e., not collected or reported to CDC); NReq = not required for school entry.

* Estimates were adjusted for nonresponse and weighted for sampling where appropriate. Estimates based on a completed vaccination series (i.e., not vaccine-specific) use the “≥” symbol. Coverage might include history of disease or laboratory evidence of immunity.

^†^ The kindergarten population is an approximation provided by each program.

^§^ Sample designs varied by state/area: census = program attempted to include all schools (public and private),and all children within schools in the assessment, and had a student response rate of ≥90%; 1-stage or 2-stage cluster sample = schools were randomly selected, and all children in the selected schools were assessed (1-stage) or a random sample of children within the schools was selected (2-stage); voluntary response = a census with a student response rate of <90% (does not imply that participation was optional).

^¶^ Some programs publish kindergarten vaccination data online that are more detailed than the state-level estimates in this table. Examples of more detailed data include county, parish, school district, and school-level estimates.

** MMR is the only measles containing vaccine available in the United States. Most states require 2 doses of MMR; Alaska, New Jersey, and Oregon require 2 doses of measles, 1 dose of mumps, and 1 dose of rubella vaccines. Georgia, New York, New York City, North Carolina, Pennsylvania, and Virginia require 2 doses of measles and mumps, 1 dose of rubella vaccines. Iowa requires 2 doses of measles and 2 doses of rubella vaccines.

^††^ Pertussis vaccination coverage might include some diphtheria, tetanus toxoids, and pertussis vaccine (DTP) vaccinations if administered in another country or by a vaccination provider who continued to use DTP after 2000. Most states require 5 doses of DTaP for school entry; Illinois, Maryland, Virginia, and Wisconsin require 4 doses; Nebraska requires 3 doses. Pennsylvania does not require pertussis vaccine. The reported coverage estimates represent the percentage of kindergartners with the state-required number of DTaP doses, except for Kentucky, which requires ≥5 but reports ≥4 doses of DTaP.

^§§^ Medians calculated from data from 48 states and the District of Columbia (i.e., does not include Oklahoma, Wyoming, Houston, New York City, Guam, Marshall Islands, Federated States of Micronesia, N. Mariana Islands, Palau, Puerto Rico, or U.S. Virgin Islands). Coverage data were reported for 3,973,172 kindergartners.

^¶¶^ The proportion surveyed likely was <100%, but is reported as 100% based on incomplete information about the actual current enrollment.

*** Did not include some types of schools, such as online schools or those located in military bases or correctional facilities.

^†††^ Kindergarten vaccination coverage data were collected from a sample, and exemption data were collected from a census of kindergartners.

^§§§^ Counted some or all vaccine doses received regardless of Advisory Committee on Immunization Practices recommended age and time interval; vaccination coverage rates reported might be higher than those for valid doses.

^¶¶¶^ For Nebraska, estimates represent coverage among children in kindergarten and first grade. For Palau, estimates represent coverage among children in first grade.

**** Reported public school data only.

^††††^ Pertussis vaccine is not required in Pennsylvania. Coverage for tetanus and diphtheria toxoids was 94.8%.

The median percentage of kindergartners with an exemption from one or more required vaccines (not limited to MMR, DTaP, and varicella vaccines) among the 46 states reporting this information was 2.0% (range = 0.1% [Mississippi] to 6.8% [Alaska]), similar to the median of 1.9% reported for this group during the 2015–16 school year (Table 2). The percentage of kindergartners with any exemption was <1% in four states (Alabama, Louisiana, Mississippi, and West Virginia), and ≥4% in nine states (Alaska, Arizona, Idaho, Maine, Nevada, Oregon, Utah, Washington, and Wisconsin). From the 2015–16 to the 2016–17 school year, the exemption rate decreased by >1.0 percentage points in two states (California and Vermont) and increased by >0.5 percentage points in seven states (Alaska, Georgia, Nevada, New Hampshire, New Mexico, North Carolina, and Wisconsin). Among states that reported exemptions by type, the median percentage of medical exemptions was 0.2% (range = <0.1% in two states [Delaware and New Mexico] to 1.5% [Alaska]), and the median percentage of nonmedical exemptions was 1.8% (range = 0.5% [DC] to 6.5% [Oregon]).

**TABLE 2:** Estimated number and percentage* of children enrolled in kindergarten with reported type of exemption from vaccination and grace period/provisional enrollment, by immunization program^†^ — United States and territories, 2016–17 school year

Immunization program	Medical exemptions, no. (%)	Nonmedical exemptions			Any exemption				Grace period/Provisional enrollment,^§^ no. (%)
		Religious, no.	Philosophical, no.	Total no. (%)	2016–2017, No.	2016–2017, %	2015–2016, %	Percentage point difference (2015–16 to 2016–17)	
**Median^¶^**	**(0.2)**	**—**	**—**	**(1.8)**	**—**	**2.0**	**1.9**	**0.1**	**(2.0)**
Alabama	62 (0.1)	367	**	367 (0.6)	429	0.7	0.8	-0.1	NA
Alaska	149 (1.5)	514	**	514 (5.2)	663	6.8	5.9	0.9	NA
Arizona	134 (0.2)	^††^	4,106	4,106 (4.9)	4,240	5.1	4.7	0.4	NA
Arkansas	24 (0.1)	169	344	513 (1.3)	537	1.4	1.3	0.1	3,014 (7.6)
California	2,928 (0.5)	^§§^	^§§^	3,217 (0.6)	6,144	1.1	2.5	-1.4	10,999 (1.9)
Colorado^¶¶^	NA	NA	NA	NA	NA	NA	4.3	NA	NA
Connecticut	107 (0.3)	701	**	701 (1.8)	808	2.1	2.0	0.1	NA
Delaware	7 (<0.1)	133	**	133 (1.2)	140	1.2	1.2	0.0	NA
District of Columbia	47 (0.6)	42	**	42 (0.5)	89	1.1	1.0	0.1	NA
Florida	841 (0.4)	4,725	**	4,725 (2.1)	5,566	2.5	2.2	0.3	7,293 (3.2)
Georgia	198 (0.1)	3,613	**	3,613 (2.7)	3,811	2.8	1.9	0.9	308 (0.2)
Hawaii	20 (0.1)	455	**	455 (2.7)	474	2.8	2.9	-0.1	310 (1.8)
Idaho	86 (0.4)	127	1,265	1,392 (6.2)	1,478	6.5	6.1	0.4	444 (2.0)
Illinois^¶¶^	NA	NA	NA	NA	NA	NA	NA	NA	NA
Indiana	112 (0.1)	697	**	697 (0.8)	809	1.0	1.2	-0.2	NA
Iowa	79 (0.2)	622	**	622 (1.6)	701	1.8	1.8	0.0	1,478 (3.7)
Kansas	115 (0.3)	569	**	569 (1.5)	683	1.8	1.6	0.2	NA
Kentucky	217 (0.4)	366	**	366 (0.7)	583	1.1	0.9	0.2	NA
Louisiana	54 (0.1)	32	364	396 (0.7)	450	0.8	0.8	0.0	NA
Maine	32 (0.2)	36	622	658 (4.8)	691	5.0	4.5	0.5	154 (1.1)
Maryland	391 (0.5)	628	**	628 (0.9)	1,019	1.4	1.3	0.1	NA
Massachusetts	191 (0.3)	702	**	702 (1.0)	893	1.3	1.3	0.0	NA
Michigan	213 (0.2)	872	3,262	4,134 (3.5)	4,347	3.7	3.6	0.1	885 (0.7)
Minnesota^¶¶^	NA	NA	NA	NA	NA	NA	NA	NA	NA
Mississippi	31 (0.1)	^††^	**	^††,^**	31	0.1	<0.1	0.1	210 (0.5)
Missouri^¶¶^	NA	NA	NA	NA	NA	NA	NA	NA	NA
Montana	53 (0.4)	391	**	391 (3.3)	444	3.7	3.8	-0.1	209 (1.7)
Nebraska***	186 (0.7)	367	**	367 (1.4)	553	2.0	2.0	0.0	881 (3.2)
Nevada	53 (0.1)	1,585	**	1,585 (4.3)	1,638	4.4	2.0	2.4	1,042 (2.8)
New Hampshire	29 (0.2)	365	**	365 (3.0)	394	3.2	2.6	0.6	633 (5.2)
New Jersey	196 (0.2)	1,881	**	1,881 (1.7)	2,077	1.9	1.8	0.1	1,191 (1.1)
New Mexico	6 (<0.1)	604	**	604 (2.2)	610	2.3	1.3	1.0	182 (0.7)
New York (including New York City)	345 (0.2)	1,975	**	1,975 (0.9)	2,320	1.0	0.9	0.1	4,444 (2.0)
New York City	78 (0.1)	581	**	581 (0.6)	659	0.6	0.4	0.2	1,444 (1.4)
North Carolina	174 (0.1)	2,073	**	2,073 (1.6)	2,247	1.8	1.1	0.7	2,138 (1.7)
North Dakota	24 (0.2)	64	244	307 (3.1)	332	3.4	3.3	0.1	NA
Ohio	414 (0.3)	^§§^	^§§^	2,836 (2.1)	3,251	2.4	2.3	0.1	6,320 (4.6)
Oklahoma^†††^	79 (0.2)	290	620	910 (1.7)	989	1.9	1.6	0.3	NA
Oregon	55 (0.1)	^§§^	^§§^	2,992 (6.5)	3,047	6.7	6.3	0.4	NA
Pennsylvania	537 (0.4)	1,256	1,523	2,778 (1.9)	3,315	2.3	2.2	0.1	11,622 (8.1)
Rhode Island	22 (0.2)	109	**	109 (1.0)	131	1.2	1.1	0.1	NA
South Carolina	55 (0.1)	1,124	**	1,124 (1.9)	1,180	2.0	1.6	0.4	385 (0.6)
South Dakota	21 (0.2)	219	**	219 (1.8)	241	2.0	1.6	0.4	NA
Tennessee	103 (0.1)	882	**	882 (1.1)	985	1.3	1.1	0.2	1,007 (1.3)
Texas (including Houston)	822 (0.2)	^§§^	^§§^	6,078 (1.6)	6,900	1.8	1.6	0.2	NA
Houston	69 (0.2)	^§§^	^§§^	333 (0.8)	401	1.0	0.9	0.1	NA
Utah	88 (0.2)	4	2,391	2,395 (4.9)	2,483	5.1	4.6	0.5	1,061 (2.2)
Vermont	15 (0.2)	234	**	234 (3.7)	249	3.9	5.7	-1.8	408 (6.4)
Virginia	225 (0.2)	1,048	**	1,048 (1.0)	1,273	1.2	1.2	0.0	NA
Washington	805 (0.9)	257	3,187	3,444 (4.0)	4,161	4.8	4.5	0.3	1,824 (2.1)
West Virginia	75 (0.3)	^††^	**	^††,^**	75	0.3	0.2	0.1	1,198 (4.2)
Wisconsin	194 (0.3)	271	3,238	3,509 (5.2)	3,702	5.5	3.3	2.2	1,567 (2.3)
Wyoming	NA	NA	NA	NA	NA	NA	NA	NA	NA
Guam	0 (<0.1)	7	**	7 (0.2)	7	0.2	<0.1	0.2	NA
Marshall Islands	0 (0.0)	0	**	0 (0.0)	0	0.0	NA	NA	NA
Federated States of Micronesia (Kosrae)	0 (0.0)	0	0	0 (0.0)	0	0.0	NA	NA	NA
Federated States of Micronesia (Yap)	0 (0.0)	0	0	0 (0.0)	0	0.0	NA	NA	NA
N. Mariana Islands	0 (0.0)	0	0	0 (0.0)	0	0.0	0.0	0.0	NA
Palau***	0 (0.0)	^§§^	^§§^	0 (0.0)	0	0.0	NA	NA	NA
Puerto Rico	43 (0.2)	57	**	57 (0.2)	101	0.4	0.3	0.1	NA
U.S. Virgin Islands	0 (<0.1)	12	**	12 (0.9)	12	0.9	0.6	0.3	NA

**Abbreviation:** NA = not available (i.e., not collected or reported to CDC).

* Estimates were adjusted for nonresponse and weighted for sampling where appropriate.

^†^ Medical exemptions, nonmedical exemptions, and grace period/provisional enrollment status might not be mutually exclusive. Some children might have both medical and nonmedical exemptions, and some enrolled under a grace period/provisional enrollment might be exempt from one or more vaccinations.

^§^ A grace period is a set number of days during which a student can be enrolled and attend school without proof of complete vaccination or exemption. Provisional enrollment allows a student without complete vaccination or exemption to attend school while completing a catch-up vaccination schedule. In states with one or both of these policies, the estimates represent the number of kindergartners within a grace period, provisionally enrolled, or some combination of these categories.

^¶^ Medians calculated from data from 45 states and District of Columbia; states/jurisdictions excluded were Colorado, Illinois, Minnesota, Missouri, and Wyoming. Houston, New York City, Guam, Marshall Islands, Federated States of Micronesia, N. Mariana Islands, Palau, Puerto Rico, and U.S. Virgin Islands were also excluded. Exemption data were reported for 3,666,870 kindergartners. Grace period/provisional enrollment median was calculated from data from 27 states; data were reported for 2,463,131 kindergartners.

** Philosophical exemptions were not allowed.

^††^ Religious exemptions were not allowed.

^§§^ Religious and philosophical exemptions were not reported separately.

^¶¶^ Program did not report the number of children with exemptions, but instead reported the number of exemptions for each vaccine, which could count some children more than once. Lower bounds of the percentage of children with any exemptions estimated using the individual vaccines with the highest number of exemptions are: for Colorado, 0.2% with medical exemptions, 0.3% with religious exemptions, 3.1% with philosophical exemptions, and 3.6% with any exemptions; for Illinois, 0.2% with medical exemptions, 1.2% with religious exemptions, and 1.4% for any exemptions; for Minnesota, 0.2% with medical exemptions, 3.1% with nonmedical exemptions, and 3.3% for any exemptions; and for Missouri 0.2% with medical exemptions, 2.0% with religious exemptions, and 2.2% for any exemptions.

*** For Nebraska, estimates represent exemptions among children in kindergarten and 1st grade. For Palau, estimates represent exemptions among children in 1st grade.

^†††^ Reported public school data only.

Twenty-seven states^¶¶¶^ reported data on grace period or provisional enrollment for the 2016–17 school year. The median reported percentage of kindergartners attending school during a grace period or provisional enrollment was 2.0% (range = 0.2% [Georgia] to 8.1% [Pennsylvania]) (Table 2). In 12 of 27 states reporting for the 2016–17 school year, the percentage of children provisionally enrolled or within a grace period at the time of the assessment exceeded the percentage of children with exemptions from one or more vaccines.

## Discussion

During the 2016–17 school year, kindergarten vaccination coverage for MMR, DTaP, and varicella vaccine each approached 95%, and the median exemption rate among children attending kindergarten was 2%; these rates have been relatively consistent since the 2011–12 school year. The median percentage of kindergartners attending school under a grace period or provisional enrollment was 2.0%. Although vaccination rates have remained high and stable, four states have reported coverage <90% for at least one vaccine for at least 6 consecutive years (*3*). In addition, coverage can vary within states, and clusters of undervaccinated kindergartners can exist in states with high overall rates.

Four states (California, New York, North Dakota, and Tennessee) reported increases in coverage of ≥1.5 percentage points for all reported vaccines (*3*); these increases might have resulted from programmatic measures to address undervaccination and incomplete documentation of vaccination during the 2016–17 school year. California eliminated new nonmedical exemptions for kindergartners attending public or private school (*4*,*5*) and continued to educate school staff members on criteria for provisional enrollment, thus reducing provisional enrollment from 4.4% to 1.9% (*6*). New York conducted webinars to train school staff members on vaccination requirements, exemptions, and exclusion policies; coverage increased by >1.5 percentage points for all reported vaccines in 2016–17 (Assessment Branch, Immunization Services Division, CDC, unpublished data, 2017). In North Dakota, school superintendents were educated about the importance of immunizations and their mandated role in enforcement of requirements (*7*), which prompted most school districts in the state to begin strict enforcement of school vaccination requirements, leading to increases in coverage of >3 percentage points for MMR, DTaP, and varicella vaccine in 2016–17. In Tennessee, the immunization program worked to increase the proportion of public school kindergartners who were completely up to date in the state’s immunization information systems and to improve schools’ capacity to correctly assess student vaccination status; MMR, DTaP, and varicella vaccination coverage increased >3 percentage points in Tennessee in 2016–17 (Assessment Branch, Immunization Services Division, CDC, unpublished data, 2017).

In 12 of 27 states reporting for the 2016–17 school year, the percentage of children provisionally enrolled or within a grace period at the time of the assessment exceeded the percentage of children with exemptions for one or more vaccines, indicating that children who do not have exemptions are not receiving their childhood immunizations in a timely fashion. The median percentage of children provisionally enrolled or within a grace period for the 2016–17 school year was 2.0%, which is the same as for the 2015–16 school year. Pennsylvania’s estimated grace period and provisional enrollment prevalence increased from 5.1% to 8.1%, probably because the assessment date changed from March 31 in the 2015–16 school year to December 31 in the 2016–17 school year, giving students enrolled under the grace period less time to complete required vaccination and documentation (Assessment Branch, Immunization Services Division, CDC, unpublished data, 2017). CDC encourages programs to collect and use these data to identify areas with high rates of provisional or grace period enrollment, where increasing coverage through a targeted intervention might be possible.

The number of states sharing local-level school vaccination coverage increased from 25 to 30 *(**8**)*. The online sharing of local-level data with the public contributes to transparency in public health by placing information about the risk for vaccine preventable diseases in the hands of parents and communities. The type of data published (exemptions, vaccine-specific coverage, complete vaccination, compliance with documentation requirements, and other information) varies across states, as does the geographic level of detail (school, school district, county, region of the state, or other geographic or administrative area), and the method of displaying the data (table, chart, map, or other format).

The findings in this report are subject to at least four limitations, which have been reported previously (*8*). First, comparability is limited because of variations in states’ requirements, data collection methods, and definitions of grace period and provisional enrollment. Second, representativeness might be negatively affected because of data collection methodologies that miss some schools or students or assess vaccination status at different times. Collecting vaccination and exemption data from a validated census of schools and students can improve comparability and representativeness of the data, and therefore, census data are the most programmatically useful. The majority of immunization programs do use a census to collect vaccination and exemption data. Third, actual vaccination coverage, exemption estimates, or both might be under- or overestimated because of improper or absent documentation. Finally, median coverage estimates include only 48 of 50 states and DC, median exemptions estimates include only 45 of 50 states and DC, and the median grace period or provisional enrollment estimate includes only 27 states for the 2016–17 school year.

Kindergarten vaccination requirements provide an opportunity for children to be fully vaccinated with recommended age-appropriate vaccines and to catch up on any missed early childhood vaccinations. CDC works with immunization programs to monitor kindergarten vaccination coverage, improve data quality, and promote data use for effective program planning. Based on state-level kindergarten vaccination data reported to CDC, median vaccination coverage was consistently high and median exemption rates were consistently low. However, clusters of low vaccination coverage continue to serve as opportunities for outbreaks of vaccine-preventable diseases (*9*). Because vaccination coverage and exemption levels are clustered locally, availability of local-level vaccination data can help immunization programs identify schools that might be vulnerable in an outbreak. CDC is working with programs to improve collection and use of grace period and provisional enrollment data to understand contributing factors for reported undervaccination and identify programmatic actions that might increase vaccination coverage among kindergartners.

**Summary**

**What is already known about this topic?**

Immunization programs conduct annual kindergarten vaccination assessments to monitor school-entry vaccination coverage for all state-required vaccines.

**What is added by this report?**

Median vaccination coverage was 94.0% for 2 doses of measles, mumps, and rubella vaccine; 94.5% for the state-required number of doses of diphtheria, tetanus, and acellular pertussis vaccine; and 93.8% for 2 doses of varicella vaccine. The median exemption level remained low (2.0%) but exemption rates varied by state. The median proportion of kindergartners under a grace period or provisional enrollment was 2.0%, the same as in 2015–16.

**What are the implications for public health practice?**

Vaccination coverage might vary at the local level. School assessment allows immunization programs to focus on schools with lower vaccination coverage and higher exemption levels, allowing schools to follow up with undervaccinated students to help ensure kindergartners are protected from vaccine preventable diseases.

## Footnotes

* Federally funded immunization programs are located in the 50 states and District of Columbia (DC) (“states”), five cities, and eight U.S territories and freely associated states (“territories”). Two cities reported data to CDC, which were included in their state data to calculate medians. Immunization programs in U.S. territories reported vaccination coverage and exemptions to CDC; however, these data were not included in median calculations.

† Median vaccination coverage was determined using estimates for 49 states; Oklahoma and Wyoming did not report data because of widespread problems with the quality of data reported by schools. Data from cities and territories were not included in median calculation.

§ Median exemption rate was determined using estimates for 46 states; Wyoming did not report data because of widespread problems with the quality of data reported by schools, and Colorado, Illinois, Minnesota, and Missouri were excluded because they could not report the number of kindergartners with an exemption because of the way they collect the data. Data from local areas and territories were not included in median calculation.

¶ Assessment date varied by state/area.

** California included data for independent study students in public school data and data for homeschools with six or more students in private school data. Massachusetts included two virtual schools in the public school data. North Dakota reported some homeschool data separately. Oregon reported some homeschool data separately; children enrolled in public online homeschools were included in the public school data. Pennsylvania included all homeschooled students in their public school data. Utah included some homeschooled students in public and private school data. Vermont included homeschooled students in their public and private school data if the students were enrolled in one or more classes at a school; homeschooled children who were exclusively homeschooled were not subject to vaccination requirements and were not included in these estimates.

†† States using a census attempted to collect data from all kindergartners at all schools and succeeded in collecting data for ≥90% of students. The type of sample employed by the nine states using a sample to collect coverage data varied and included a stratified two-stage cluster sample (eight states) and a stratified one-stage cluster sample (one state). A voluntary response of schools was defined as a census survey with a response rate <90% of the known population of kindergartners. A mix of methods included two or more described sampling methods, usually a census for one school type and voluntary response for the other.

§§ All 50 states and DC required 2 doses of a measles-containing vaccine; MMR is the only measles-containing vaccine available in the United States. Local DTaP requirements varied. Nebraska required 3 doses, four states (Illinois, Maryland, Virginia, and Wisconsin) required 4 doses, Pennsylvania did not require pertussis vaccination, and all other states required 5 doses, unless the fourth dose was administered on or after the fourth birthday. The reported coverage estimates represent the percentage of kindergartners with the state-required number of DTaP doses, except for Kentucky, which required 5 doses of DTaP by age 5 years, but reported 4-dose coverage for kindergartners. Eight states required 1 dose of varicella vaccine and 42 states and DC required 2 doses.

¶¶ Alabama, Florida, Georgia, Iowa, Mississippi, New Hampshire, and New Jersey considered kindergartners up-to-date only if they had received all doses of all vaccines required for school entry.

*** Most immunization programs that used census or voluntary response provided CDC with data aggregated at the state or local (city or territory) level. Coverage and exemption data based on a census or voluntary response were adjusted for nonresponse using the inverse of the response rate, stratified by school type (public, private, and home school, where available). Programs that used complex sample surveys provided CDC with deidentified data aggregated at the school or county level for weighted analysis. Weights were calculated to account for sample design and adjusted for nonresponse for data collected through complex sample design wherever possible.

††† The kindergarten population is an approximation provided by each immunization program. The totals reported here are the summations of the kindergarten population among programs reporting data for coverage, exemptions, and grace period or provisional enrollment. Data from cities and territories were not included in these totals.

§§§ https://www.cdc.gov/vaccines/imz-managers/coverage/schoolvaxview/data-reports/index.html.

¶¶¶ Twenty-seven states (Arkansas, California, Florida, Georgia, Hawaii, Idaho, Iowa, Maine, Michigan, Mississippi, Montana, Nebraska, Nevada, New Hampshire, New Jersey, New Mexico, New York, North Carolina, Ohio, Pennsylvania, South Carolina, Tennessee, Utah, Vermont, Washington, West Virginia, and Wisconsin) reported data on kindergartners attending school under a grace period or provisional enrollment.
