# *Erratum*: Vol. 66, No. 40

**DOI:** 10.15585/mmwr.mm6642a10

**Published:** 2017-10-27

In the report “Vaccination Coverage for Selected Vaccines, Exemption Rates, and Provisional Enrollment Among Children in Kindergarten — United States, 2016–17 School Year,” on page 1074 the first sentence of footnote “^§§^” should have read “**All 50 states and DC required 2 doses of a measles-containing vaccine.**”

On page 1077, in Table 1, footnote “**” should have read “**Most states require 2 doses of MMR; Alaska, New Jersey, and Oregon require 2 doses of measles, 1 dose of mumps, and 1 dose of rubella vaccines. Georgia, New York, New York City, North Carolina, Pennsylvania, and Virginia require 2 doses of measles and mumps, and 1 dose of rubella vaccines. Iowa requires 2 doses of measles and 2 doses of rubella vaccines.**”

